# Genetic transitions in the Neolithic and Bronze Age at Mas d’en Boixos (Catalonia, Spain)

**DOI:** 10.1016/j.isci.2025.112871

**Published:** 2025-06-11

**Authors:** Xavier Roca-Rada, Daniel R. Cuesta-Aguirre, Diana C. Vinueza-Espinosa, Roberta Davidson, Shyamsundar Ravishankar, Leonard Taufik, Núria Armentano, Xavier Esteve, Yassine Souilmi, João C. Teixeira, Assumpció Malgosa, Bastien Llamas, Cristina Santos

**Affiliations:** 1 Australian Centre for Ancient DNA, School of Biological Sciences, University of Adelaide, Adelaide, SA, Australia; 2 Faculty of Arts and Humanities, University of Coimbra, Coimbra, Portugal; 3 Unitat Antropologia Biològica and GREAB – Grup de Recerca en Antropologia Biològica, Universitat Autònoma de Barcelona, Barcelona, Spain; 4 Indigenous Genomics, The Kids Research Institute Australia, Adelaide, SA, Australia; 5 Mochtar Riady Institute for Nanotechnology, Tangerang, Indonesia; 6 Museu d’Arqueologia de Catalunya, Barcelona, Spain; 7 Servei de Patrimoni Arqueològic i Paleontològic-Generalitat de Catalunya, ERAAUB, IAUB, Barcelona, Spain; 8 Environment Institute, University of Adelaide, Adelaide, SA, Australia; 9 National Centre for Indigenous Genomics, Australian National University, Canberra, ACT, Australia; 10 Centre of Excellence for Australian Biodiversity and Heritage (CABAH), University of Adelaide, Adelaide, SA, Australia; 11 Evolution of Cultural Diversity Initiative, Australian National University, Canberra, ACT, Australia; 12 Centre for Interdisciplinary Studies, University of Coimbra, Coimbra, Portugal

**Keywords:** Human genetics, Biological sciences, Paleobiology

## Abstract

Mas d’en Boixos is a key prehistoric site in Northeastern Iberia spanning from the Early Neolithic to the Late Iron Age. We analyzed genome-wide data from eight individuals and ten mitogenomes, dated to the Middle Neolithic and Early Bronze Age, alongside three previously published Iron Age individuals. Two Middle Neolithic individuals buried together were first-degree maternal relatives and carried Western Hunter-Gatherer, Anatolian, and residual Magdalenian-associated ancestries. Conversely, six Early Bronze Age individuals buried in a hypogeum exhibited distinct mitochondrial lineages. Among them, three were third-degree relatives, and all males shared a Y-chromosome lineage, consistent with a collective burial of an extended family within a patrilocal society practicing possible female exogamy. These individuals showed genetic continuity with additional Steppe-related ancestry, which displayed a subtle southward gradient across Iberia. We also identified an Eastern European mitochondrial lineage—challenging the proposed male-driven Bronze Age transition—and Mediterranean gene flow—suggesting dynamic interactions across the sea.

## Highlights

• Two Neolithic relatives carried WHG, Anatolian and residual Magdalenian-related ancestries
• A Bronze Age hypogeum reflects a patrilocal family burial with possible female exogamy
• Steppe-related ancestry showed a subtle southward gradient in Bronze Age Iberia
• Mediterranean gene flow indicates dynamic Bronze Age interactions across the sea

## Graphical abstract

**Figure:**
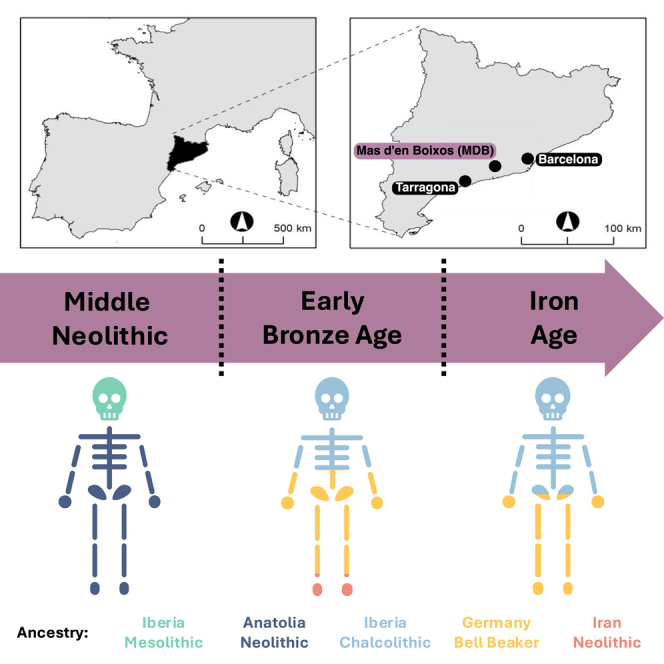

## Introduction

**Figure 1:**
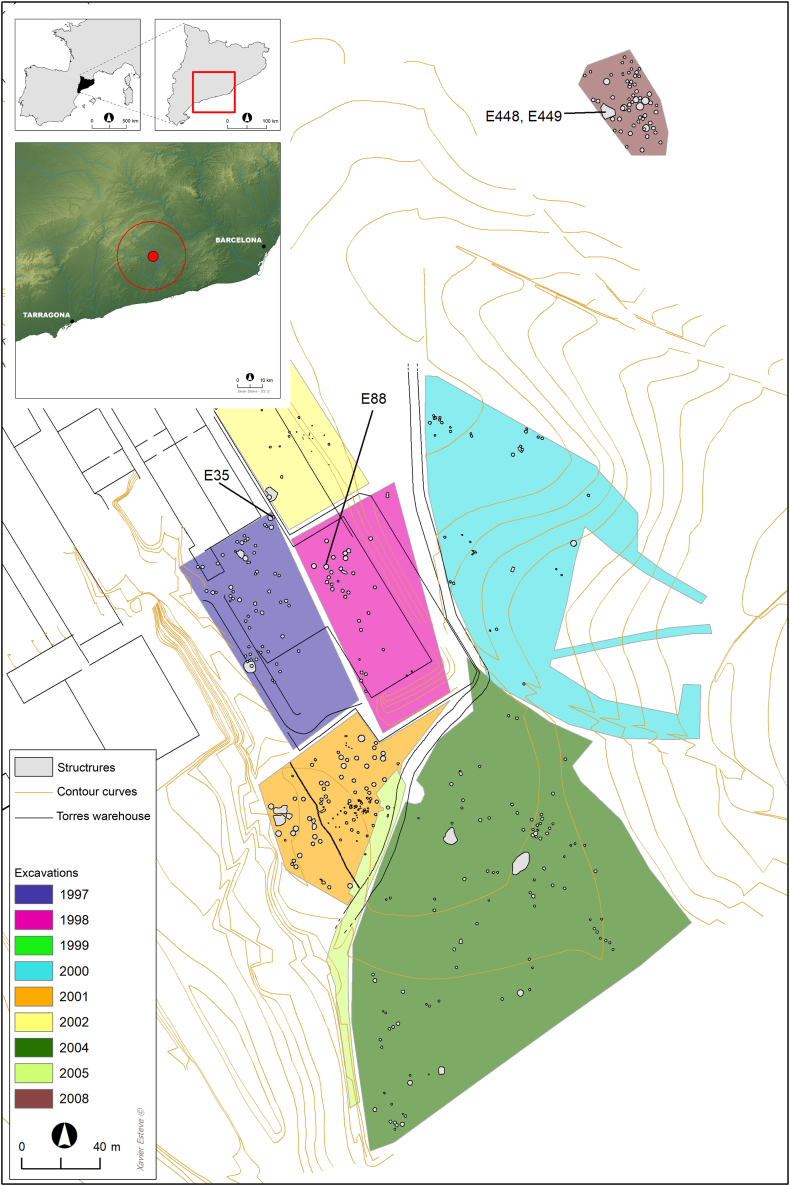
Location and topography of Mas d’en Boixos in the Catalan pre-littoral depression of the Penedès region (Barcelona, Catalonia, Spain) and the positions of the archaeological structures discussed in this study

Mas d’en Boixos (MDB) is arguably the most significant prehistoric archaeological site in the Catalan pre-littoral depression of the Penedès region (Barcelona, Catalonia, Spain) (Figure 1).^1^^,^^2^^,^^3^ Since excavations began in 1997, more than 450 structures have been uncovered, revealing a rich and complex stratigraphy that spans from the Early Neolithic to the Late Iron Age, approximately 7,500 to 2,200 years ago (7.5–2.2 ka).^1^^,^^2^^,^^3^

MDB is particularly notable for its diverse array of burial practices and structural types. These include silo-type graves, characterized by their ellipsoid and cylindrical shapes, as well as hypogeal chambers that served as burial sites for multiple individuals.^1^^,^^2^^,^^3^ Although the grave goods associated with these burials are often limited, they provide valuable insights into the social practices and cultural norms of each period.^1^^,^^2^^,^^3^

The site’s temporal span aligns with significant cultural and genetic transitions across Europe. The first of these is the Neolithic transition, which marked a shift from hunting and gathering to agriculture, leading to the establishment of settled communities.^4^^,^^5^^,^^6^^,^^7^^,^^8^^,^^9^ This transformation was largely driven by population movements from Anatolia (present-day Türkiye) into Europe ∼8.5 ka,^5^ leading to dynamic interactions between farmers and local hunter-gatherers.^5^^,^^10^^,^^11^^,^^12^^,^^13^^,^^14^^,^^15^^,^^16^^,^^17^^,^^18^ Notably, Mesolithic hunter-gatherers from Northern and Northeastern Iberia retained a smaller proportion of Last Glacial Maximum (LGM) ancestry associated with the Magdalenian culture (Goyet_Q2 cluster) compared to those from other regions of the peninsula, being most of their ancestry more closely linked to post-LGM expansions (Villabruna/Oberkassel cluster).^11^^,^^12^^,^^16^^,^^19^ Recent genetic studies have revealed that Neolithic Iberian populations exhibited a notable persistence of diverse Mesolithic hunter-gatherer—higher than that observed in Central Europe—due to admixture along migration routes and additional local contributions.^10^^,^^11^^,^^12^

The second major transition, occurring ∼5 ka, is the Bronze Age transition, characterized by migrations of Pontic Steppe herders into North and Central Europe.^20^ This migration not only shifted the genetic ancestry of North and Central Europeans but also had an impact on the genetic makeup of Iberians, introducing substantial Steppe-related ancestry.^11^^,^^13^^,^^20^^,^^21^^,^^22^^,^^23^^,^^24^^,^^25^^,^^26^ In Northeastern Iberia (current Catalonia, Spain), burial practices from this period have sparked debates about the relationships among individuals buried in close proximity. While some scholars argue that these relationships suggest nuclear family ties^27^^,^^28^, others propose a more complex social structure, emphasizing the possibility of extended family connections or cultural affiliations.^28^^,^^29^^,^^30^^,^^31^^,^^32^

By the Iron Age (∼2.8–2 ka), Iberian populations retained significant proportions of Steppe-related ancestry, but regional differences became more pronounced. While some groups maintained genetic continuity with Bronze Age populations, others experienced subtle shifts, either through increased Steppe-related ancestry or influences from the Mediterranean.^11^

**Figure 2:**
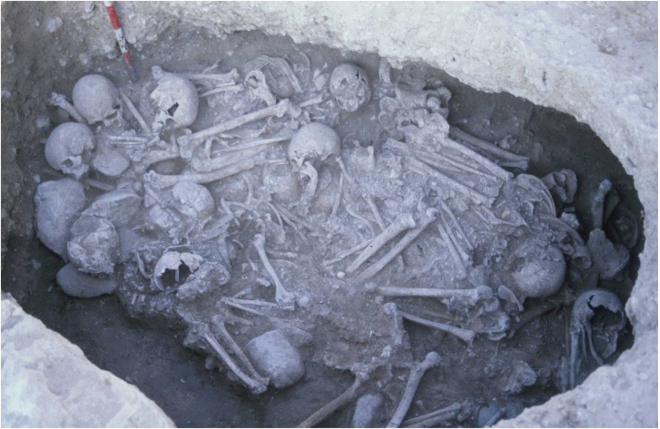
The Early Bronze Age hypogeum (E35) from MDB

In this study, 25 new individuals from MDB were genetically analyzed. Two Middle Neolithic individuals (MDB_MN) associated with the post-Cardial culture were recovered from the same burial (E88) (Figure 1), while 23 Early Bronze Age individuals (MDB_EBA) were unearthed from a hypogeum (E35) (Figures 1 and 2). This hypogeum consisted of a single cavity accessed through a vertical pit and was used exclusively for successive funerary purposes.^1^ The scarcity of associated artifacts hinders precise cultural determination, but the discovery of a small bronze fragment aligns with the time period. Additionally, we included three Iron Age individuals (MDB_IA) from two structures (E448 and E449) that were previously analyzed by Olalde et al.^11^

Using paleogenomic data from MDB, this study aims to: (1) infer kinship relationships among individuals buried in close proximity; (2) explore diachronic genetic changes at MDB; and (3) contextualize the findings within the broader genetic history of Iberia and Europe.

## Results

### Genetic sex, uniparental markers, and kinship in MDB

The two individuals from the Middle Neolithic burial were genetically identified as one male and one female (Table S4). They were first-degree relatives (Table S8) and shared the same mitochondrial haplotype, belonging to K1a4a1 haplogroup (Table S7), suggesting they could be full siblings or a mother and son. While the male individual carried the Y-chromosome haplogroup I-Y21979 (I2a1a1b2∼; Tables S5 and S17), no upstream-derived SNPs within haplogroup I were recovered; therefore, the assignment to I-Y21979 should be considered tentative.

In the Early Bronze Age hypogeum, genome-wide data was successfully recovered for six out of the 23 individuals analyzed (Table S1), consisting of four males and two females (Table S4). Additionally, eight mitochondrial genomes were reconstructed, each belonging to a distinct haplogroup: H3, R0a, H1e1a, H1bm, X2b, U2e1, U5b1, and H1c (Table S7). However, all males carried Y-chromosome subhaplogroups within the R-M269 (R1b1a1b) lineage (Tables S5, S14–16, and S18). While three could be assigned to more derived subclades—R-P312 (R1b1a1b1a1a2), R-A6484 (R1b1a1b1a1a2c1a5c5), and R-Y37285 (R1a1a1b1a1a1a2b∼)—overall haplogroup resolution was limited due to low coverage and assignments should be interpreted with caution.

Kinship analyses revealed that most Early Bronze Age individuals were unrelated, except for three who shared third-degree relationships among themselves: a female (MDB_24215) and two males (MDB_24233 and MDB_24197) (Table S8). We also observed that the average kinship coefficient of unrelated male pairs was higher than that of unrelated female and male-female pairs (Table S13). Only two individuals from MDB_EBA passed quality thresholds for ROH analysis and none of their profiles suggested consanguinity or small population size (Figure S1).

Finally, kinship analysis of the two males and one female from the Iron Age revealed that they were unrelated (Table S8). They all carried different mitochondrial haplogroups (H, H3, and J1c1) and the two males had different sub-haplogroups from the R-M269 lineage, concretely R-P312.^11^

### Genetic affinities within MDB

**Figure 3:**
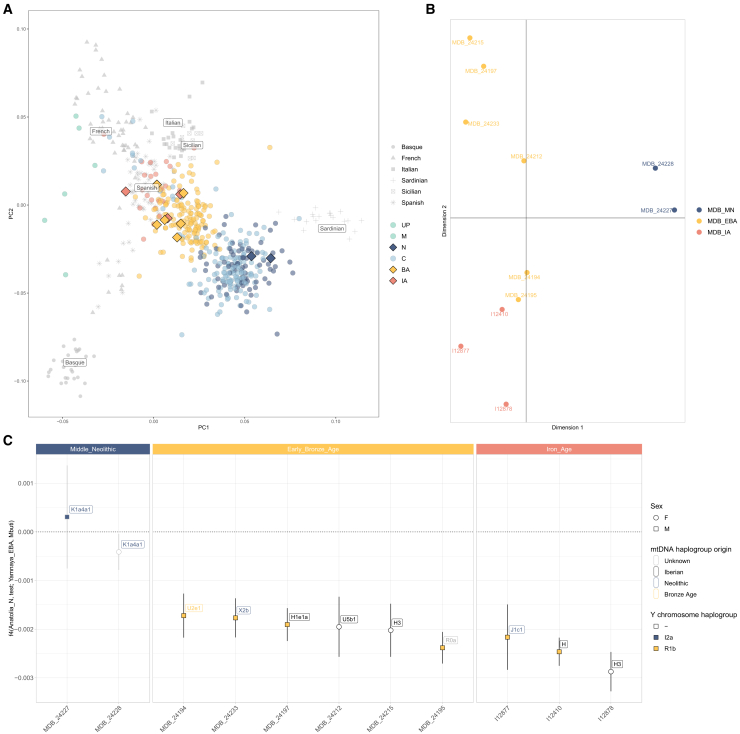
Genetic structure and ancestry transitions in ancient Iberia and MDB (A) PCA of present-day Western Europeans (light gray) with projected ancient Iberians as colored circles (Upper Palaeolithic (UP) and Mesolithic (M) Iberian Hunter-Gatherers, N: Neolithic; C: Chalcolithic; BA: Bronze Age; and IA: Iron Age), including the MDB individuals as colored diamonds. (B) Iterations of outgroup *f*_3_-statistics of the form *f*_3_ (Mbuti; Ind1, Ind2) visualized in a multidimensional scaling analysis of 1 - *f*_3_. (C) *f*_4_-statistics are displayed as the mean ± 1-SD with gray bars representing *Z* scores ≤3 and black bars *Z* scores ≥3. Results show an increased affinity to Russia_Samara_EBA_Yamnaya (absent in MDB_MN) which implies the presence of Steppe-related ancestry in MDB_EBA and MDB_IA. This plot also shows the turnover in Y-chromosome lineages in male individuals (color-filled squares) and the origin of their mtDNA haplogroups.

To explore genetic affinities among MDB individuals over time, iterations of outgroup *f*_3_-statistics of the form *f_3_*(Mbuti; Ind1, Ind2) were conducted and visualized using a multidimensional scaling analysis of 1 - *f*_3_ (Figure 3B). The results revealed a distinct genetic grouping for MDB_MN, distinguishing them from others. Notably, the Early Bronze Age hypogeum individuals who shared third-degree relationships (MDB_24215, MDB_24233, and MDB_24197) clustered closely together. The remaining Early Bronze Age individuals displayed a gradual genetic shift toward the three Iron Age individuals.

### Neolithic transition in MDB and Iberia

When examining genetic affinities between MDB and ancient Iberians projected into a PCA with present-day Southwestern Europeans (Figure 3A), MDB_MN clustered with Iberian individuals from the Neolithic and Chalcolithic, located between present-day Basques and Sardinians.

**Figure 4:**
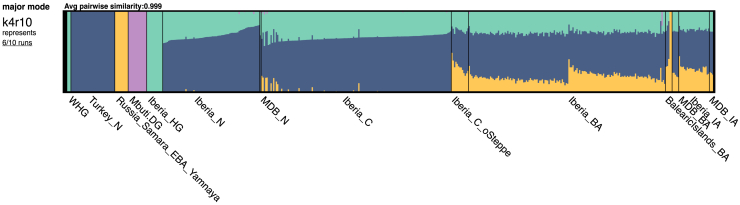
Ancestry proportions estimated with a supervised ADMIXTURE at k = 4, with the four predefined ancestries being Western Hunter-Gatherers (WHG), Turkish individuals from the Neolithic representing the Anatolian Farmers ancestry (Anatolia_N), Bronze Age Eurasians from the Yamnaya culture representing the Steppe-related ancestry (Russia_Samara_EBA_Yamnaya) and present-day African individuals being proxy for Sub-Saharan ancestry (Mbuti) with ancient Iberians from the pre-Neolithic (hunter-gatherers; HG), Neolithic (N), Chalcolithic (C), Bronze Age (BA) and Iron Age (IA)

**Figure 5:**
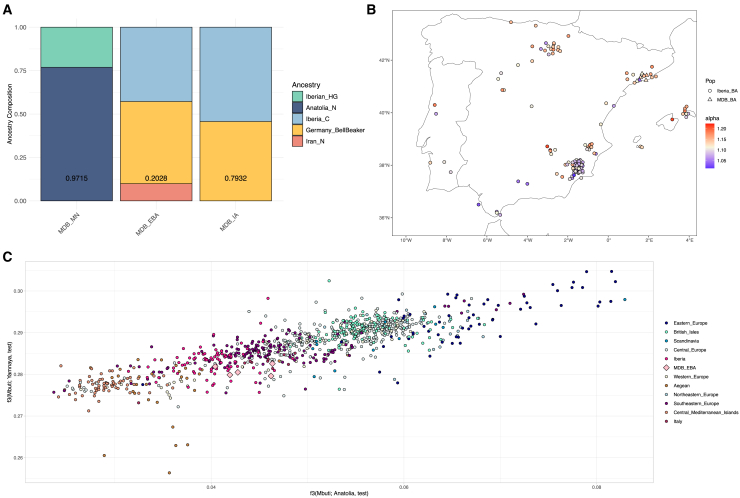
Steppe-related ancestry in ancient Iberia and MDB (A) Ancestry proportions for MDB temporal groups using *qpAdm*; *p* values are provided inside each column. (B) The Russia_Samara_EBA_Yamnaya ancestry proportion in Iberia during the Bronze Age computed with *f*_4_-ratios colored from highest to lowest using a color scale. The MDB individuals are represented as triangles. The Russia_Samara_EBA_Yamnaya ancestry proportion decreases southwards. (C) Outgroup *f*_3_-statistics of the form *f*_3_(Mbuti; Pop, X) showing the amount of shared drift between the ancient individuals (X) with Turkish individuals from the Neolithic representing the Anatolian Farmers ancestry (Anatolia_N; x-axis) and Bronze Age Eurasians from the Yamnaya culture representing the Steppe ancestry (Russia_Samara_EBA_Yamnaya; y-axis). Clusters of individuals are represented in shades of blue, pink, and amber from the top-left to the bottom-right. The MDB individuals are represented as colored diamonds.

The ADMIXTURE analysis (k = 4; Figures 4 and S2) suggests that MDB_MN had an admixed genetic ancestry comprising a pre-Neolithic Western Hunter-Gatherer (WHG) component and a Neolithic ancestry component from Anatolia (Anatolia_N), similar to the rest of Neolithic and Chalcolithic Iberia. These signals were further confirmed through *qpAdm* ancestry modeling, which showed that MDB_MN could be modeled as ∼77% Anatolia_N and ∼23% Iberian_HG (*p* = 0.9715; Table S10 and Figure 5A).

To investigate the hunter-gatherer ancestry of MDB_MN, outgroup *f*_*3*_-statistics of the form *f*_*3*_(MDB_MN, Test; Mbuti), where *Test* rotates through different Iberian_HG, was computed. In this analysis, we observed that MDB_MN shared a higher genetic drift with the Southeastern Iberian Mesolithic hunter-gatherer from Cingle_del_Mas_Nou (València) and to the Central Portuguese Late Mesolithic hunter-gatherer from Moita_do_Sebastião, despite low *Z* scores. These were followed by Los_Canes and La_Braña (Northern Iberia) with higher *Z* scores (Figure S3). These individuals are more closely related to post-LGM hunter-gatherers than Magdalenian-related hunter-gatherers. Additionally, *qpAdm* ancestry modeling using WHG (Luxembourg_Loschbour and Hungary_EN_HG_Koros) instead of Iberian_HG as a source, did not necessarily require Goyet_Q2 as an additional third component (*p* = 0.2118), even though it improved the model fit (*p* = 0.7751; Table S10).

### Bronze Age transition in MDB, Iberia, and Western Eurasia

All Early Bronze Age and Iron Age individuals from MDB showed a genetic shift from the Neolithic and Chalcolithic pattern observed in PCA. Concretely, MDB_EBA and MDB_IA clustered with Iberian individuals from the Bronze and Iron Age periods, moving toward present-day Spanish (Figure 3A).

The Steppe-related ancestry, represented by Russia_Samara_EBA_Yamnaya, was present in most Iberians from the Bronze and Iron Ages, including MDB_EBA and MDB_IA, as evidenced by ADMIXTURE analysis (Figure 4) and *f*_4_-statistics of the form *f*_4_(Anatolia_N, test; Russia_Samara_EBA_Yamnaya, Mbuti), where *Test* iterated through each MDB individual (Figure 3C).

Admixture modeling of a distal framework with WHG, Anatolia_N and Russia_Samara_EBA_Yamnaya as sources did not explain the genetic ancestry of MDB_EBA (Table S11), however it fitted MDB_IA (*p* = 0.1367), and the model improved when including Iberian_HG as an additional fourth source (*p* = 0.8057; Table S12). While semiproximal frameworks that included Iberia_C (instead of WHG and Anatolia_N) as well as Russia_Samara_EBA_Yamnaya or Germany_BellBeaker also explained the genetic ancestry of MDB_IA (*p* = 0.7599 and *p* = 0.7932, respectively; Table S12 and Figure 5A), these models still failed for MDB_EBA (Tables S11). Instead, MDB_EBA was best fitted by three-source models (Table S11). When using Iberia_C and Russia_Samara_EBA_Yamnaya, the best-fitting models included Italy_C and Iran_N as additional sources (*p* = 0.2986 and *p* = 0.1408, respectively). Alternatively, when using Iberia_C and Germany_BellBeaker, the best-fitting models included Iran_N and Iran_C (*p* = 0.2028 and *p* = 0.1525, respectively; Figure 5A).

To explore a more proximal framework, we used Iberia_C_oSteppe (Northern Iberians from the Chalcolithic representing the earliest evidence of Steppe-related ancestry in the Peninsula), which provided insight into the genetic variation of MDB_EBA (Table S11). The best-fitting models included: (1) Iran_N as the most distal additional source (*p* = 0.1974); (2) Italy_C and Levant_C as the most semiproximal (*p* = 0.4657 and *p* = 0.2283, respectively); and (3) CentralMediterraneanIslands_BA and Jordan_BA as the most proximal (*p* = 0.2420 and *p* = 0.2414, respectively). These affinities with Central and Eastern Mediterranean populations were further supported by *f*-statistics (Figure S4). Interestingly, MDB_IA was solely explained with Iberia_C_oSteppe (Table S11).

We further investigated the Steppe-related ancestry component in Bronze Age Iberians using *f*_4_-ratios of the form of *f*_4_(Russia_Samara_EBA_Yamnaya, Mbuti; test, Morocco_Iberomaurusian)/*f*_4_(Russia_Samara_EBA_Yamnaya, Mbuti; Anatolia_N, Morocco_Iberomaurusian), excluding individuals with absolute *Z* scores higher than twice their standard error. Most *f*_4_-ratios exceeded 1, indicating, as expected, higher Russia_Samara_EBA_Yamnaya ancestry than Anatolia_N relative to Morocco_Iberomaurusian. A nuanced decrease in Russia_Samara_EBA_Yamnaya ancestry was observed southwards in Iberia (Figure 5B), being higher near the Pyrenees and lower in Southern Iberia.

To explore the Bronze Age transition in Iberia relative to other European populations, two outgroup *f*_3_-statistics per individual were computed: *f*_3_(Mbuti; Anatolia_N, Test) on the x-axis and *f*_3_(Mbuti; Russia_Samara_EBA_Yamnaya, Test) on the y-axis (Figure 5C), revealing a north-south cline of clusters. A cluster including individuals from the British Isles, Scandinavia, Northeastern and Eastern Europe had the highest shared drift with Russia_Samara_EBA_Yamnaya (shades of blue). This cline was followed by Central and Western Europeans with wide distributions (light gray and beige). An extended cluster with a pronounced cline included Southeastern Europeans, Italians, and Iberians (shades of pink), including diverse MDB_EBA individuals. The closest cluster to Iberia comprised Mediterranean and Aegean individuals (shades of amber).

## Discussion

### Middle Neolithic burial

While the anthropological study identified two females in the Middle Neolithic burial associated with the post-Cardial culture (E88), genetic analyses revealed one male and one female. These individuals were first-degree relatives and shared the same mitochondrial haplotype associated to the K1a4a1 haplogroup. Given the evidence for simultaneous or closely timed burials, the anthropological description suggests that they were likely siblings or a mother-son pair, as these appear to be the most parsimonious explanations.

The genetic profile of MDB_MN aligns with previous research on the Iberian Peninsula, indicating admixture between local hunter-gatherers and Anatolian farmers.^11^^,^^12^^,^^13^^,^^19^^,^^24^ This conclusion is further supported by analysis of uniparentally-inherited markers. In Iberia, other Neolithic individuals have been found carrying the K1a4a1 subhaplogroup.^10^^,^^11^^,^^21^^,^^26^^,^^33^^,^^34^ Interestingly, the same subhaplogroup—but a slightly different haplotype—was found in a Chalcolithic sample from the Paris Street archaeological site in Cerdanyola del Vallès,^26^ only ∼52 km away from MDB. In fact, it has been suggested that the mitochondrial haplogroup K1a probably reached the European continent ∼8 ka during migrations of Early Neolithic Southwestern Asians through continental and Mediterranean routes.^33^^,^^35^^,^^36^^,^^37^

The Middle Neolithic male from MDB carried the I-Y21979 (I2a1a1b2∼) Y-chromosome subhaplogroup. It has been suggested that I-L460 (I2a1) lineages were already present among Central European hunter-gatherers^21^^,^^38^^,^^39^^,^^40^ and were later assimilated by the aforementioned wave of Eastern Mediterranean Neolithic farmers moving westward, who were associated with mitochondrial haplogroup K1a and Y-chromosome haplogroup G-P15 (G2a). In fact, Southeastern and Southwestern European farmers from the Starčevo culture—which gave rise to the Linear Pottery culture (LBK)—and the Cardial Ware culture carried both I-L460 (specifically, I-P37.2/I2a1a) and G-P15 lineages.^7^^,^^19^^,^^21^^,^^35^^,^^38^^,^^39^^,^^41^^,^^42^ Interestingly, Olalde et al. (2015)^19^ suggested that populations related to the Cardial and Linear Pottery cultures were descended from a common farming population in the Balkans, which had subsequently migrated further westwards into Europe along the Mediterranean coast and Danube river. The Cardial-related individuals were found to be most closely related to present-day Sardinians and Basques.^19^ Indeed here, the nuclear genetic information of the studied post-Cardial individuals from MDB corroborates this affinity.

Finally, the low persistence of Upper Palaeolithic Magdalenian ancestry in MDB_MN aligns with similar findings from continental Europe, as well as Northern and Northeastern Iberia.^11^^,^^12^^,^^13^^,^^16^^,^^19^ However, further sampling of Upper Palaeolithic, Mesolithic, and Neolithic Iberian populations is necessary to fully understand the genetic landscape of the Iberian hunter-gatherer ancestry over time.

### Early Bronze Age hypogeum and the Bronze Age transition in Iberia

In Northeastern Iberia, the Bronze Age is marked by distinct burial practices between coastal and mountainous regions. Coastal sites such as MDB are primarily characterized by pit graves and hypogea, reflecting collective burial practices typical of lowland and coastal areas. In contrast, mountainous regions, like Montanissell,^30^ are associated with megalithic constructions such as dolmens, alongside the use of natural features like caves and caverns for burials. These patterns align with broader trends observed across Europe, where coastal areas often feature simpler burial practices, while mountainous regions are characterized by monumental and collective funerary structures, emphasizing the influence of geography on social and ritual practices. It has been previously hypothesized that individuals found in these collective burials—regardless of whether they were part of caves, pits, or more complex funerary structures—were part of nuclear family ties.^27^^,^^28^ However, an extended family structure unifying kinship and culture in small societies was suggested in Montanisell^30^ and might also be the case in MDB.

Eight individuals with diverse mitochondrial haplogroups and low levels of consanguinity were identified. Notably, all males likely shared the same Y-chromosome lineage. Additionally, three third-degree relationships were detected, involving one female and two males. We also observed that the average kinship coefficient among unrelated male pairs was higher than that of unrelated female and male-female pairs. These findings suggest an extended kinship network and align with the possibility of a patrilocal society characterized by possible female exogamy, a pattern previously observed in Southeastern Iberia during the Bronze Age.^43^

Most of the MDB_EBA individuals carried maternal lineages (H1, H3, and U5) that were previously reported for Neolithic and Chalcolithic Iberians and probably expanded throughout Europe after the LGM from the Iberian/Franco-Cantabrian refugium.^44^^,^^45^^,^^46^^,^^47^ Furthermore, one individual carried a lineage that arrived in Iberia during the Neolithic transition (X2b)^10^ and while little is known about the subhaplogroup H1bm, it has been found in Turkmenistan_EN,^48^ Germany_LN^48^ and Spain_BA^48^ and Crete_BA.^49^ Notably, MDB_ 24194 carried a previously unreported mtDNA linage in ancient Iberia, U2e1, that has higher affinity to Palaeolithic/Mesolithic Eastern Hunter-Gatherers (EHG) and Bronze Age Yamnaya culture, but not to Neolithic farmers.^50^^,^^51^ Additionally, haplogroups H1b and H1c, also present in MDB_EBA, are commonly found in Eastern European populations.^52^^,^^53^ Hence, these maternal lineages indicate a degree of Steppe-related affinity in MDB_EBA.

Furthermore, all MDB_EBA males carried Y-chromosome subhaplogroups within the R-M269 (R1b1a1b) lineage, which is prevalent in individuals associated with the Steppe Yamnaya culture and associated with the spread of some Indo-European languages.^21^ In Iberia, it has been suggested that there was a Y-chromosome turnover during the Bronze Age with the arrival of the Steppe-related ancestry,^11^^,^^25^^,^^26^ which we also observed when comparing MDB_EBA from MDB_MN.

In fact, Martiniano et al.^24^ analyzed three Middle Bronze Age Portuguese males who carried R-M269 Y-chromosome haplogroups and Neolithic Iberian maternal lineages (H1+152, U5b3, and X2b+226), similar to some individuals from the MDB_EBA group. However, these individuals exhibited markedly lower Steppe-related ancestry compared to populations in Central and Northern Iberia. A similar pattern was observed in some Southeastern Iberians reported by Villalba-Mouco et al..^25^ Martiniano et al.^24^ proposed that Indo-European R-M269 males entered Iberia around 3.8 ka as a small group of migrants, forming familial relationships with local females and gradually reducing their Steppe-related ancestry with each generation. Within a few centuries, this process resulted in only trace levels of Steppe-related ancestry. Our findings also reveal a subtle southward gradient in Steppe-related ancestry across Iberia, with higher levels near the Pyrenees and diminishing further south. Notably, Iberia exhibits some of the lowest Steppe-related ancestry in Europe, surpassed only by Italy and the Mediterranean islands.

Even though the Bronze Age transition has been suggested to have been male driven,^11^ some mitochondrial lineages associated with Eastern European populations have been detected in MDB_EBA, suggesting that females were probably migrating as well. In fact, Villalba-Mouco et al.^25^ did not find any signal for a male-biased Steppe-related ancestry in Bronze Age Iberia.

Finally, the R0a mtDNA haplogroup was found in an MDB male and it was reported for the first time in ancient Iberia by Villalba-Mouco et al..^25^ In present-day populations, this haplogroup is more frequent in the Arabian Peninsula, but it has also been found in ancient individuals from the Eastern Mediterranean, as well as Ukraine Yamnaya and Sicilian Bell Beakers. While Gandini et al.^54^ suggested that it might represent a relic of Late Glacial or postglacial dispersals around the Mediterranean, Villalba-Mouco and colleagues suggested that the Mediterranean region was a route of dissemination during the Chalcolithic and Early Bronze Age as R0a has not been found in Central Europe. Moreover, Villalba-Mouco and colleagues also detected an excess of Iran_N-like ancestry in populations from the Chalcolithic and Early Bronze Age in Southeastern Iberia in the same way as other Chalcolithic and Bronze Age Eastern and Central Mediterranean populations,^25^ suggesting a Mediterranean influence in the peninsula. Interestingly, the only suitable frameworks in admixture modeling for MDB_EBA included Mediterranean populations as an additional source, further supporting this Mediterranean influence at least before or during the Early Bronze Age period in Northeastern Iberia, contrasting with its absence in MDB_MN.

### IA burials

Nowadays, Spaniards and Portuguese carry higher Steppe ancestry proportions than Bronze Age Iberians meaning that other posterior migrations took place after the Bronze Age. The two males and the female previously analyzed by Olalde et al.^11^ had different mitochondrial haplogroups (H, H3, and J1c1), were unrelated and the males had R-M269 Y-chromosome haplogroups. The three individuals were relatively distinct from each other and closer to the Early Bronze Age individuals than the Middle Neolithic individuals. These three individuals as well as the rest of the previously published Spanish Iron Age individuals generally had a higher Steppe-related ancestry than previous periods. Interestingly, the Iran_N-like ancestry was not found in these individuals, indicating a lack of Mediterranean influence during this period and a dissipation of such ancestry over the generations in MDB. However, it has to be noted that the small sample size of only three individuals from the site may not be representative enough to draw definitive conclusions. Actually, little is known about Iron Age Iberia from a genetic standpoint, as the most common funerary practice was cremation,^55^ which is not favorable for DNA preservation. Hence, it is still not known if the previously published Spanish Iron Age individuals (*n* = 24) are representative of the main Iron Age Iberian population.

### Limitations of the study

Despite the comprehensive genetic analysis performed, this study has some limitations. The relatively low coverage for some male individuals restricted the resolution of Y-chromosome haplogroup assignments and limited the ability to detect finer-scale kinship structure. Additionally, the scarcity of associated grave goods and contextual archaeological information for some burials makes precise cultural interpretations challenging. Finally, although the sample size is substantial for the site, it may not fully capture the broader genetic diversity or demographic dynamics of the wider region during these periods.

## Resource availability

### Lead contact

Further information and requests for resources should be directed to and will be fulfilled by the lead contact: Cristina Santos (Cristina.Santos@uab.cat).

### Materials availability

This study did not generate new reagents.

### Data and code availability

• Data: The raw sequencing data and the processed aligned sequences are available through the Sequence Read Archive (SRA), BioProject: PRJNA1244027; the genotype dataset is available as a Data S1.
• Code: This paper does not report original code.
• Other items: The dataset of previously published ancient and modern individuals^56^ is accessible at https://reich.hms.harvard.edu/datasets.

## Acknowledgments

Part of the laboratory work was conducted at 10.13039/501100001786The University of Adelaide with support from Holly Heininger, Vilma Pérez, Corinne Mensforth, Navdeep Kaur, and Gemma Harvey. Computational analyses were conducted using supercomputing resources provided by the Phoenix HPC service at the University of Adelaide. The 10.13039/501100004837Spanish Ministry of Science and Innovation supported the project (PID2022-136748NB-I00, “Demographic and Social Organization, Mobility, Kinship, and Disease during the Bronze Age: The Challenge of Collective Burials (ColBAB)”) through the Proyectos de Generación del Conocimiento 2022. The 10.13039/100007615Australian Research Council though BL Future Fellowship (FT170100448). We sincerely thank the two anonymous reviewers for their insightful and constructive comments.

## Author contributions

Conceptualization and design: C.S., A.M., and B.L.; archaeological and anthropological contextualization: N.A. and X.E.; laboratory work: X.R.-R., D.C.V.-E., L.T., and B.L.; data analysis: X.R.-R., R.D., D.R.C.-A., and S.R.; interpretation: all authors; manuscript writing: X.R.-R.; and manuscript editing: all authors.

## Declaration of interests

The authors declare no competing interests.

## STAR★Methods

### Key resources table

**Table:** 

REAGENT or RESOURCE	SOURCE	IDENTIFIER
**Biological samples**		

Ancient skeletal element	This study	MDB_24195, LP110_3, 103_E35
Ancient skeletal element	This study	MDB_24197, LP110_5, 463A_E35
Ancient skeletal element	This study	MDB_24194, LP110_2, 216_E35
Ancient skeletal element	This study	MDB_24233, LP110_26, IND_8_E35
Ancient skeletal element	This study	MDB_24212, LP110_6, 701_E35
Ancient skeletal element	This study	MDB_24215, LP110_9, 104_E35
Ancient skeletal element	This study	MDB_24227, LP110_21, IND_1_E88
Ancient skeletal element	This study	MDB_24228, LP110_22, IND_2_E88
Ancient skeletal element	This study	MDB_24213, LP110_7, 640_E35
Ancient skeletal element	This study	MDB_24193, LP110_1, 82_E35
Ancient skeletal element	This study	MDB_24216, LP110_10, 462_E35
Ancient skeletal element	This study	MDB_24214, LP110_8, 350_E35
Ancient skeletal element	This study	MDB_24231, LP110_24, IND_19_E35
Ancient skeletal element	This study	MDB_24223, LP110_17, 264_E35
Ancient skeletal element	This study	MDB_24232, LP110_25, IND_20_E35
Ancient skeletal element	This study	MDB_24221, LP110_15, 666_E35
Ancient skeletal element	This study	MDB_24218, LP110_12, 74_E35
Ancient skeletal element	This study	MDB_24230, LP110_23, IND_12_E35
Ancient skeletal element	This study	MDB_24224, LP110_18, IND_10_E35
Ancient skeletal element	This study	MDB_24219, LP110_13, 463B_E35
Ancient skeletal element	This study	MDB_24217, LP110_11, 278_E35
Ancient skeletal element	This study	MDB_24196, LP110_4, 279_E35
Ancient skeletal element	This study	MDB_24222, LP110_16, IND_11_E35
Ancient skeletal element	This study	MDB_24225, LP110_19, 329_E35
Ancient skeletal element	This study	MDB_24220,LP110_14, 193_E35

**Chemicals, peptides, and recombinant proteins**		

T4 DNA ligase	Life Technologies	Cat#EL0012
UltraPure™ DNase/RNase-Free Distilled Water	Life Technologies	Cat#10977023
USER enzyme	E-Freezer	Cat#M5505L
UGI	E-Freezer	Cat#M0281L
T4 PNK	E-Freezer	Cat#M0201L
T4 DNA Polymerase	E-Freezer	Cat#M0203L
BST DNA Polymerase	E-Freezer	Cat#M0275L
Tango Buffer (10X)	Life Technologies	Cat#BY5
dNTP set (100mM), 8 x 1.25mL	Life Technologies	Cat#10297117
RSA (Albumin from rabbit serum)	Sigma	Cat#A0639-5G
10 mM ATP	E-Freezer	Cat#P0756L
KAPA HiFi HotStart ReadyMix	Roche	Cat#7958935001
Brilliant III Ultra-Fast SYBR Green ROX qPCR Master Mix	Integrated Sciences	Cat#600892
Ethanol for molecular biology,200 proof (absolute)	Thermo Fisher	Cat#FSBBP2818-500
Herculase II Fusion DNA Polymerase	Integrated Sciences	Cat#600677
Platinum™ *Taq* DNA Polymerase High Fidelity	Thermo Fisher	Cat#11304029
1 M Tris-HCI Buffer ph 7.5	Thermofisher	Cat#15567027
2x Hi-RPM Hybridization Buffer (25mL)	Integrated Sciences	Cat#5190-0403
Fisher Scientific Tween™ 20, Fisher BioReagents™	Thermo Fisher	Cat#FSBBP337100
EDTA, 0.5M, pH 8.0	Life Tech	Cat#AM9262
Sodium Acetate	Sigma	Cat#S7899
EZ-Link™ Psoralen-PEG3-Biotin	Life Technologies	Cat#29986
DMSO	Sigma	Cat#D8418
Glycogen RNA grade 20mg/ml	Life Technologies	Cat#R0551
Dnase I	E-freezer	Cat#M0303S
Polyethylene glycol 8000 (PEG 8000)	VWR	Cat#0159-500G
Taq DNA Polymerase with ThermoPol® Buffer	E-freezer	Cat#M0267S
EDTA solution pH 8.0 (0.5 M) for molecular biology	PanReac AppiChem	Cat#A4892,1000
Guanidine Hydrochloride *BioChemica*	PanReac AppiChem	Cat#A1499,1000
2-Propanol	PanReac AppiChem	Cat#131090.1611
Sodium acetate, 3M, pH 5.2	Thermo Scientific	Cat#J61928.AK
Tween® 20	PanReac AppiChem	Cat#A4974,0500
Tris-HCl, 1 M, pH 8,0	Thermo Scientific	Cat#J22638.AE

**Critical commercial assays**		

myBaits Expert Human Affinities Prime Plus Kit	Arbor Biosciences	N/A
Twist Ancient DNA reagent (TE-94002772)	Twist Bioscience	Cat#101042
Twist Mitochondrial Panel	Twist Bioscience	Cat#104562
Twist Binding and Purification Beads kit	Twist Bioscience	Cat#100984
Twist Hybridization and Wash Kit	Twist Bioscience	Cat#101026
Twist Wash Buffers kit	Twist Bioscience	Cat#100846
MinElute Reaction Cleanup Kit (250) ERC buffer	Qiagen	Cat#28206
D1000 ScreenTapes (For 112 samples)	Integrated Sciences	Cat#5067-5582
AxyPrep Mag PCR clean up Kit	Fisher Biotec	Cat#AX-MAG-PCR-CL-50
Qubit™ 1X dsDNA HS Assay Kit	Thermo Fisher	Cat#Q33231
Roche Expand Long Range dNTPack kit	Sigma	Cat#4829034001
Rneasy Minelute reaction cleanup kit (50)	Qiagen	Cat#74204
HiScribe™ T7 High Yield RNA Synthesis Kit	E-freezer	Cat#E2040S
NEBNext Magnesium RNA Fragmentation Module	GeneSearch	Cat#E6150S
High Pure Viral Nucleic Acid Large Volume Kit	Roche Life Science	Cat#05114403001

**Deposited data**		

Sequencing data	This study	Sequence Read Archive (SRA) - BioProject: PRJNA1244027
Genotype data	This study	Data S1
Allen Ancient DNA Resource (AADR) v54.1	Mallick et.^56^	https://reich.hms.harvard.edu/allen-ancient-dna-resource-aadr-downloadable-genotypes-present-day-and-ancient-dna-data

**Oligonucleotides**		

IS1 adapter: A^∗^C^∗^A^∗^C^∗^TCTTTCCCTACACG ACGCTC	Meyer et al.^57^	IDT
IS2 adapter: G^∗^T^∗^G^∗^A^∗^CTGGAGTTCAG ACGTGTGCT	Meyer et al.^57^	IDT
IS3 adapter: A^∗^G^∗^A^∗^T^∗^CGGAA^∗^G^∗^A^∗^G^∗^C	Meyer et al.^57^	IDT
IS6: CAAGCAGAAGACGGCATACGA	Meyer et al.^57^	IDT
IS5: AATGATACGGCGACCACCGA	Meyer et al.^57^	IDT
IS7: ACACTCTTTCCCTACACGAC	Meyer et al.^57^	IDT
IS8: GTGACTGGAGTTCAGACGTGT	Meyer et al.^57^	IDT
M655-H2698-tailT7 (fwd): AATT GTAATACGACTCACTATAGGGT TGACCTGCCCGTGAAGAGG	N/A	IDT
M656-L8351 (rev): TTGGGGCATTTCA CTGTAAAGAGG	N/A	IDT
M657-H7801-tailT7 (fwd): AATTGTAA TACGACTCACTATAGGGCTA TCCTGCCCGCCATCATC	N/A	IDT
M658-L14267 (rev): GAGGGGT CAGGGTTGATTCG	N/A	IDT
M698-H13305 (fwd): TCGGCA TCAACCAACCACAC	N/A	IDT
M699-L3407-tailT7 (rev): AATTG TAATACGACTCACTATAGGGT ACAACGTTGGGGCCTTTGC	N/A	IDT

**Software and algorithms**		

bcl2fastq v2.19.1	N/A	https://support.illumina.com/content/dam/illumina-support/documents/downloads/software/bcl2fastq/bcl2fastq-2-19-1-release-notes-1000000035330-00.pdf
nf-core/eager v2.4.5	Fellows Yates et al.^58^	https://github.com/nf-core/eager
Nextflow v21.03.0.edge	Di Tommaso et al.^59^	https://www.nextflow.io/docs/latest/
FastQC v0.11.9	https://github.com/s-andrews/FastQC	N/A
MultiQC v1.13.dev0	Ewels et al.^60^	https://github.com/MultiQC/MultiQC
AdapterRemoval v2.3.2	Schubert et al.^61^	https://github.com/MikkelSchubert/adapterremoval
fastP v0.20.1	Chen^62^	https://github.com/OpenGene/fastp
BWA v0.7.17-r1188	Li et al.^63^	https://github.com/lh3/bwa
circulargenerator v1.0	Peltzer et al.^64^	https://github.com/apeltzer/CircularMapper/tree/master
Samtools v1.12	Danecek et al.^65^	http://www.htslib.org
DeDup v0.12.8	Peltzer et al.^64^	https://github.com/apeltzer/DeDup
sequenceTools v1.4.0.6	https://github.com/stschiff/sequenceTools	N/A
DamageProfiler v0.4.9	Neukamm et al.^66^	N/A
bamUtil v1.0.15	https://github.com/statgen/bamUtil	N/A
angsd v0.935	Rasmussen et al.^67^	N/A
sexdeterrmine v1.1.2	Lamnidis et al.^68^	N/A
Picard v2.26.0	http://broadinstitute.github.io/picard/index.html	N/A
Yleaf	Ralf et al.^69^^,^^70^	https://github.com/genid/Yleaf
Geneious v2022.1.1	https://www.geneious.com	N/A
Haplocart	Daniel et al.^70^	https://github.com/grenaud/vgan/wiki/HaploCart
HaploCheck version 1.3.2	Weissensteiner et al.^71^	https://github.com/genepi/haplocheck
READv2	Alaçamlı et al.^72^	https://github.com/GuntherLab/READv2
hapROH version 0.64	Ringbauer et al.^73^	https://github.com/hringbauer/hapROH
eigensoft 7.2.1	https://github.com/DReichLab/EIG	N/A
ADMIXTOOLS v5.1 & v7.0.2	https://github.com/dReichLab/AdmixTools	N/A
plink 1.9	Purcell et al.^74^	http://pngu.mgh.harvard.edu/purcell/plink/
pong 1.5	Aaron et al.^75^	https://github.com/ramachandran-lab/pong

### Experimental model and study participant details

The two Middle Neolithic individuals found in the same burial (E88) were anthropologically classified as females, with an estimated age at death of 25–35 years.^76^ Archaeologically, they were associated to the post-Cardial culture and dated to 3,715–3,638 cal BCE (4,908 ± 33 BP) and 3,810–3,696 cal BCE (4,994 ± 33 BP).

For the Early Bronze Age hypogeum, different bones were used for the determination of the minimum number of individuals, as most of the skeletal remains were intermingled and their preservation was poor. A total of 24 skulls and one-sided humerus bones were found and based on the morphological traits of the skulls, a total of 9 and 8 individuals were assigned as male and female, respectively, with the biological sex of the remaining individuals being undetermined. Out of the 23 mandibles retrieved, 18 were identified as adults and 5 as subadults.^76^ Two skeletal remains, identified as the earliest and latest burials in the hypogeum, were dated to 1,452–1,222 cal BCE (3,095 ± 50 BP) and 1,772–1500 cal BCE (3,350 ± 60 BP), respectively.

While human remains from the Iron Age are scarce at MDB, the three specimens previously reported^11^ are particularly exceptional, as they represent inhumation burials—distinct from the cremation practices commonly associated with Iberian cultures, though other traditions coexisted in the peninsula during this period.^55^ Olalde et al. (2019)^11^ analysed two individuals from one burial (E448): a male with an estimated age at death of 15-25 and dated to 517–380 cal BCE (2,350 ± 30 BP, Beta-495153) and a 6-year-old whose biological sex was not determined with the same indirect date. Olalde and colleagues also analysed another female from another burial (E449) dated to 490–364 cal BCE (2,340 ± 30 BP, Beta-495155) and with an estimated age at death of 35–45.

All dates are reported with the 95.4% CI calibrated radiocarbon age alongside the Conventional Radiocarbon Age. Calibration was performed in Calib 8.20 (http://calib.org/calib/calib.html) using the IntCal20 calibration curve. Previously, Olalde et al. (2019) reported dates calibrated with IntCal13^77^; here, we provide the newly calibrated dates using IntCal20.^78^

### Method details

#### Ancient DNA laboratory work

Ancient DNA analysis was conducted in two different clean-room facilities— the University of Adelaide’s Australian Centre for Ancient DNA (ACAD), in Australia; and the Autonomous University of Barcelona (UAB), in Spain—following strict precautions to minimise contamination.^79^ The samples were processed while wearing face masks, visors, hooded coverall, hair net, and gloves, in laboratories exclusively dedicated to ancient DNA analysis equipped with positive air flow pressure. Standard precautions to avoid contaminations were employed.^80^

A total of 25 well-preserved teeth were included in this study. Bone powder was extracted from cementum, which is richer in DNA than dentin,^81^ and DNA was extracted using a method optimised to retrieve degraded ancient DNA fragments at UAB.^82^ Partially UDG-treated double-stranded DNA libraries were generated^83^ at ACAD. All post-amplification lab work was performed in ACAD’s post-PCR facilities. Paired-end shotgun sequencing using an Illumina NovaSeq (200 cycles) was performed by service providers at the Kinghorn Centre for Clinical Genomics (Sydney, Australia).

#### Shotgun sequencing data processing

Raw data from the shotgun sequencing run underwent processing using the aDNA analysis workflow package nf-core/eager^58^ version 2.4.5 with default settings for pre-mapping processing. Merged read mates were aligned to the human reference genome (GRCh37d5) using *bwa aln* with parameters *-l 1024 -n 0.01 -o 2.*^84^ A mapping quality threshold of 25 was applied and duplicated reads were removed with markduplicates (https://broadinstitute.github.io/picard/). DamageProfiler^66^ was used to assess aDNA authenticity, calculating fragment size distributions and post-mortem damage rates at the read termini (Table S1. Shotgun sequencing metrics, Table S2. Deep shotgun sequencing metrics, Table S3. Twist capture sequencing metrics). We also calculated endogenous DNA proportions after mapping, filtering and deduplication (Table S1. Shotgun sequencing metrics, Table S2. Deep shotgun sequencing metrics, Table S3. Twist capture sequencing metrics). The nuclear contamination function from ANGSD^67^ was used to calculate the Maximum Likelihood and Method of Moments contamination estimates according to Method 1 and 2 (Tables S1 and S2). For further contamination estimates, see “Mitochondrial DNA” section.

#### Deep shotgun sequencing and library enrichment

Out of the 25 samples, 7 met the quality thresholds of having an endogenous DNA content higher than 0.1% and displaying misincorporation patterns characteristic of aDNA above ∼10% (Table S1). These samples underwent deep shotgun sequencing and were processed as previously described (Table S2). Additionally, these 7 samples, along with 8 others (a total of 15), met the quality thresholds of an endogenous DNA content higher than 0.05% and misincorporation patterns characteristic of aDNA above 3% (Table S1). These 15 samples underwent enrichment.

Libraries were firstly enriched using an in-house mtDNA enrichment protocol.^85^ Subsequently, they were enriched using two different assays for genome-wide capture. First, the Prime Plus enrichment kit (Daicel Arbor Biosciences), which includes DNA baits targeting ∼1.2 million nuclear SNPs and the whole mitochondrial genome. However, after discovering the allelic bias of this assay,^86^ we repeated enrichment with the Twist Biosciences enrichment kit, which includes DNA baits targeting the same ∼1.2 million nuclear SNPs as well as additional SNPs and tiling regions^87^ (Table S3). In the final analyses, mtDNA sequences captured with the Prime Plus were used as they are not impacted by the allelic bias discovered in the Prime Plus enrichment kit.^86^ For genome-wide analyses, only the 1.2 million nuclear SNPs captured with the Twist reagent were used, as they are less impacted by allelic bias than the Prime Plus and exhibit approximately similar levels of bias to the widely-accepted 1240k enrichment assay.^87^^,^^88^^,^^89^ The libraries were sequenced on a NovaSeq 6000 platform at the Kinghorn Centre for Clinical Genomics (Sydney, Australia).

#### Ancient genomic data processing

Sequencing raw data underwent processing as previously described. Retained reads were trimmed 2 bp from each end using the trimBam function of bamUtil (https://github.com/statgen/bamUtil) and pseudohaploid variant calling was executed using the Twist Bioscience “Twist Ancient DNA'' SNP panel^87^ with pileupCaller (https://github.com/stschiff/sequenceTools).

After filtering individuals with: (i) more than 15,000 SNPs covered by at least one read of the Twist SNPs panel (Table S3); (ii) the presence of the misincorporation patterns characteristic of aDNA (>3%) (Table S1. Shotgun sequencing metrics, Table S2. Deep shotgun sequencing metrics, Table S3. Twist capture sequencing metrics); and (iii) absence of contamination (Tables S1, S2, and S7); 8 samples for genome-wide data analyses were retained: Middle Neolithic (*n* = 2) and Early Bronze Age (*n* = 6).

### Quantification and statistical analyses

#### Genetic sex determination

Genetic sex determination was assigned using SexDetERRmine (https://github.com/nf-core/modules/tree/master/modules/nf-core/sexdeterrmine) with default quality cut-off values for -q30 and -Q30 using the screening and deep shotgun sequencings (Table S4).

#### Y-chromosome

We classified Y-chromosome haplogroups in male samples using *Yleaf*^69^ with the parameters -r 1 -q 30 -dh -p on the processed BAM files. The output was manually curated to assign the most derived haplotype. We filtered for derived alleles, excluded C→T and G →A substitutions, and selected the position with the highest depth and supported by additional upstream markers when possible. The ISOGG 2019 nomenclature was used for the assignments. Both the *Yleaf* output and the manually curated haplogroup assignments are presented in Table S5. The curated lists of derived alleles can be found in Tables S14, S15, S16, S17, and S18.

#### Mitochondrial DNA

The analysis of mtDNA involved merging raw data obtained from four distinct sources: screening and deep shotgun sequencing, an in-house mtDNA enrichment protocol,^85^ the myBaits Expert Human Affinities Prime Plus Kit by DAICEL Arbor Biosciences (Ann Harbor, MI, USA), and the Twist Bioscience “Twist Ancient DNA” reagent. Raw data underwent processing as previously described. Merged reads were then mapped to the mitochondrial revised Cambridge Reference Sequence (rCRS) using CircularMapper (https://github.com/apeltzer/CircularMapper). After read trimming and filtering following the procedures outlined above, a total of 10 samples were retained for mtDNA analyses (Table S6). The read pileups were visually inspected in Geneious v2022.1.1 (Biomatters; https://www.geneious.com) and mtDNA haplogroup calling was carried out using haplocart,^70^ and contrasted using mitoverse HaploCheck version 1.3.2, which also estimated contamination levels (Table S7).

#### Kinship analysis

READv2^72^ was used to assess kinship relationships between pairs of individuals from each time period. We selected five Mid-Late Neolithic, ten Early Bronze Age, and five Iron Age individuals to establish a baseline of unrelated individuals. These individuals were chosen based on data generated in a similar manner (captured and partially repaired) from previous publications^11^ (Table S8). Average kinship coefficients of unrelated and related pairs for the Early Bronze Age hypogeum were calculated based on the READv2^72^ results (Table S13).

#### Runs of homozygosity

We used hapROH version 0.64 with default settings to identify runs of homozygosity within the genome.^73^ Only individuals with > 400,000 SNPs covered in the panel were used in this analysis.

#### Population genetic analysis

##### Dataset

We merged our final dataset with previously published datasets of ancient and modern individuals reported by the Reich Lab^56^ (https://reich.hms.harvard.edu/datasets; please see Table S9 for a detailed list of samples and new labels used in population genetics analysis).

##### Principal component analysis (PCA)

PCA was computed using the smartpca program v10210 (EIGENSOFT) with the lsqproject and SHRINKMODE option YES and an extended list of modern and ancient populations from Eurasia, Africa and the Caucasus. The ancient individuals were projected onto PC1 and PC2.

##### *f*-statistics

To infer genetic affinities between populations, *f*_3_-statistics, *f*_4_-statistics and *f*_*4*_-ratios were computed using the qp3Pop, qpDstat with the activated f4-mode functions and qpF4ratio, respectively, implemented in ADMIXTOOLS v7.0.2 package (https://github.com/DReichLab).^90^

##### Admixture modeling

Related individuals were excluded from admixture modelling analyses. ADMIXTURE 1.3 was used to define the main genetic cluster profiles.^32^ Data was pruned for linkage disequilibrium in PLINK with parameters --indep-pairwise 50 10 0.1 and --geno 0.999.^74^ Four populations (WHG, Anatolia_N, Russia_Samara_EBA_Yamnaya and Mbtui) were selected as fixed source groups of ancestry (k = 4) implementing a supervised ADMIXTURE model^32^ and the model was replicated ten times.

We used *qpAdm* from the ADMIXTOOLS v5.1 package (https://github.com/DReichLab), with the “allsnps: YES” option to minimally reduce the number of SNPs used and subsequently increase the power to reject models, to model the ancestry in our new reported individuals. We quantified the proportion of genetic ancestry contributed by each source. The ancestry proportions in the target population are inferred on the basis of how the target population is differentially related to a set of reference/outgroups via the source populations. For all the models applied here, we have used a set of 12 outgroups (Mbuti.DG, Ethiopia_4500BP.DG, Papuan.DG, Belgium_UP_GoyetQ116_1, Czech_Vestonice, Italy_North_Villabruna_HG, Russia_MA1_HG.SG, Russia_Ust_Ishim_HG.DG, Malalmuerzo).

## Supplemental information

Document S1. Figures S1–S4

Table S1. Shotgun sequencing metrics

Table S2. Deep shotgun sequencing metrics

Table S3. Twist capture sequencing metrics

Table S4. Sex assign

Table S5. Y chromosome haplogroup determination

Table S6. mtDNA captures sequencing metrics

Table S7. mtDNA haplogroup determination

Table S8. Kinship analysis

Table S9. Labels used in population genetic analysis

Table S10. qpAdm results for MDB_MN

Table S11. qpAdm results for MDB_EBA

Table S12. qpAdm results for MDB_IA

Table S13. Average kinship coefficients of MDB_EBA pairs

Table S14. MDB_24194: curated list of derived Y chromosome alleles (Yleaf)

Table S15. MDB_24195: curated list of derived Y chromosome alleles (Yleaf)

Table S16. MDB_24197: curated list of derived Y chromosome alleles (Yleaf)

Table S17. MDB_24227: curated list of derived Y chromosome alleles (Yleaf)

Table S18. MDB_24233: curated list of derived Y chromosome alleles (Yleaf)

Data S1. Genotype dataset in Eigenstrat format
