# Supplementary material for: Genetic transitions in the Neolithic and Bronze Age at Mas d’en Boixos (Catalonia, Spain)
Source: iScience. 2025 Jun 11;28(7):112871. doi: 10.1016/j.isci.2025.112871 (PMC12273517; doi:10.1016/j.isci.2025.112871)
Supplement: Document S1. Figures S1–S4 [file mmc1.pdf]

iScience, Volume *28*

Supplemental information

Genetic transitions in the Neolithic and Bronze Age at Mas d'en Boixos (Catalonia, Spain)

Xavier Roca-Rada, Daniel R. Cuesta-Aguirre, Diana C. Vinueza-Espinosa, Roberta Davidson, Shyamsundar Ravishankar, Leonard Taufik, Núria Armentano, Xavier Esteve, Yassine Souilmi, João C. Teixeira, Assumpció Malgosa, Bastien Llamas, and Cristina Santos

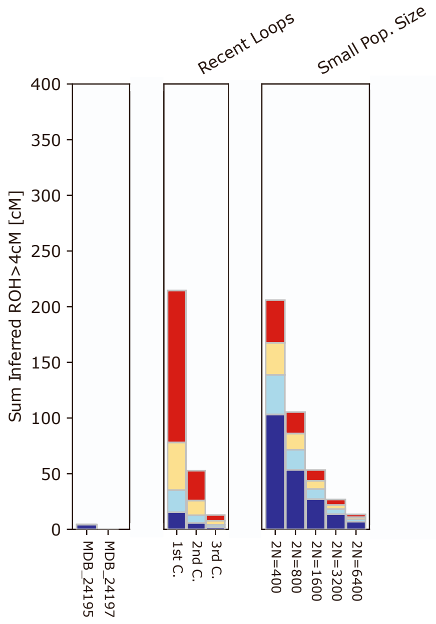

Figure S1. Runs of Homozygosity (ROH) analysis for individuals from the present study, exhibiting cumulative ROH lengths exceeding 4 cM and coverage of more than 400,000 SNPs from the 1240k dataset.

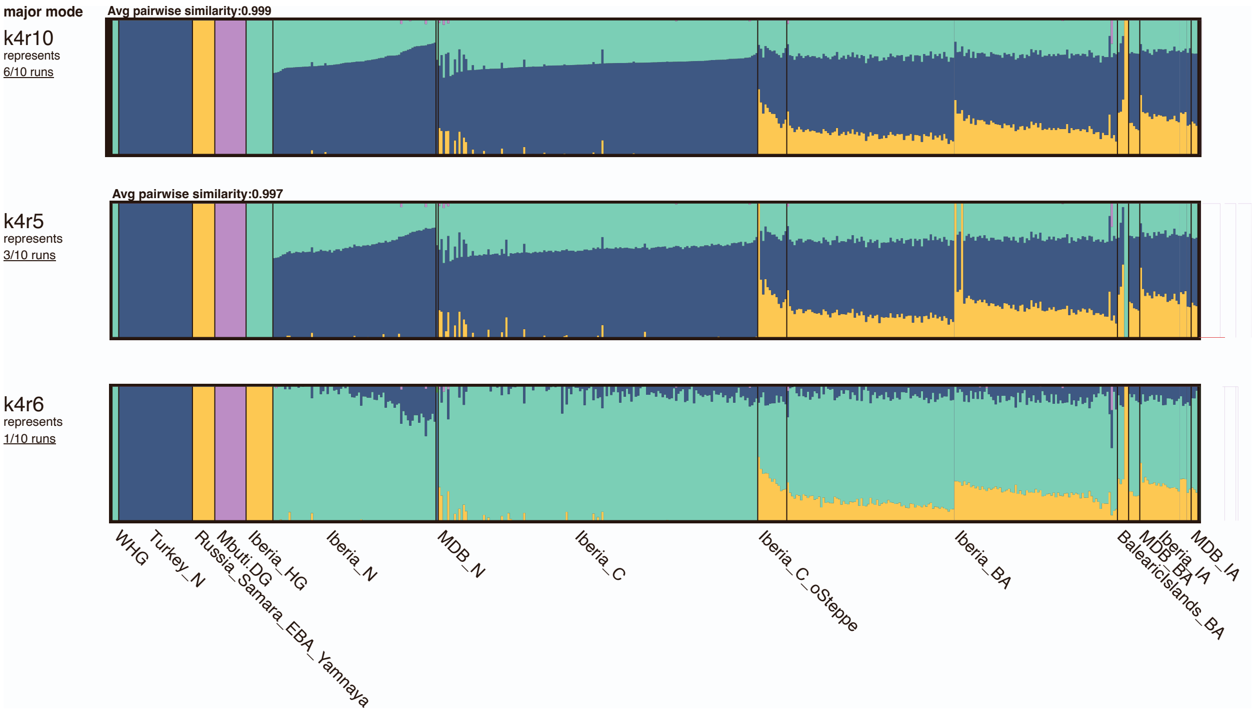

Figure S2. Ancestry proportions estimated with a supervised ADMIXTURE at k=4, with the four predefined ancestries being Western Hunter-Gatherers (WHG), Turkish individuals from the Neolithic representing the Anatolian Farmers ancestry (Anatolia_N), Bronze Age Eurasians from the Yamnaya culture representing the Steppe ancestry (Russia_Samara_EBA_Yamnaya) and present-day African individuals being proxy for Sub-Saharan ancestry (Mbuti) with ancient Iberians from the pre-Neolithic (hunter-gatherers; HG), Neolithic (N), Chalcolithic (C), Bronze Age (BA) and Iron Age (IA).

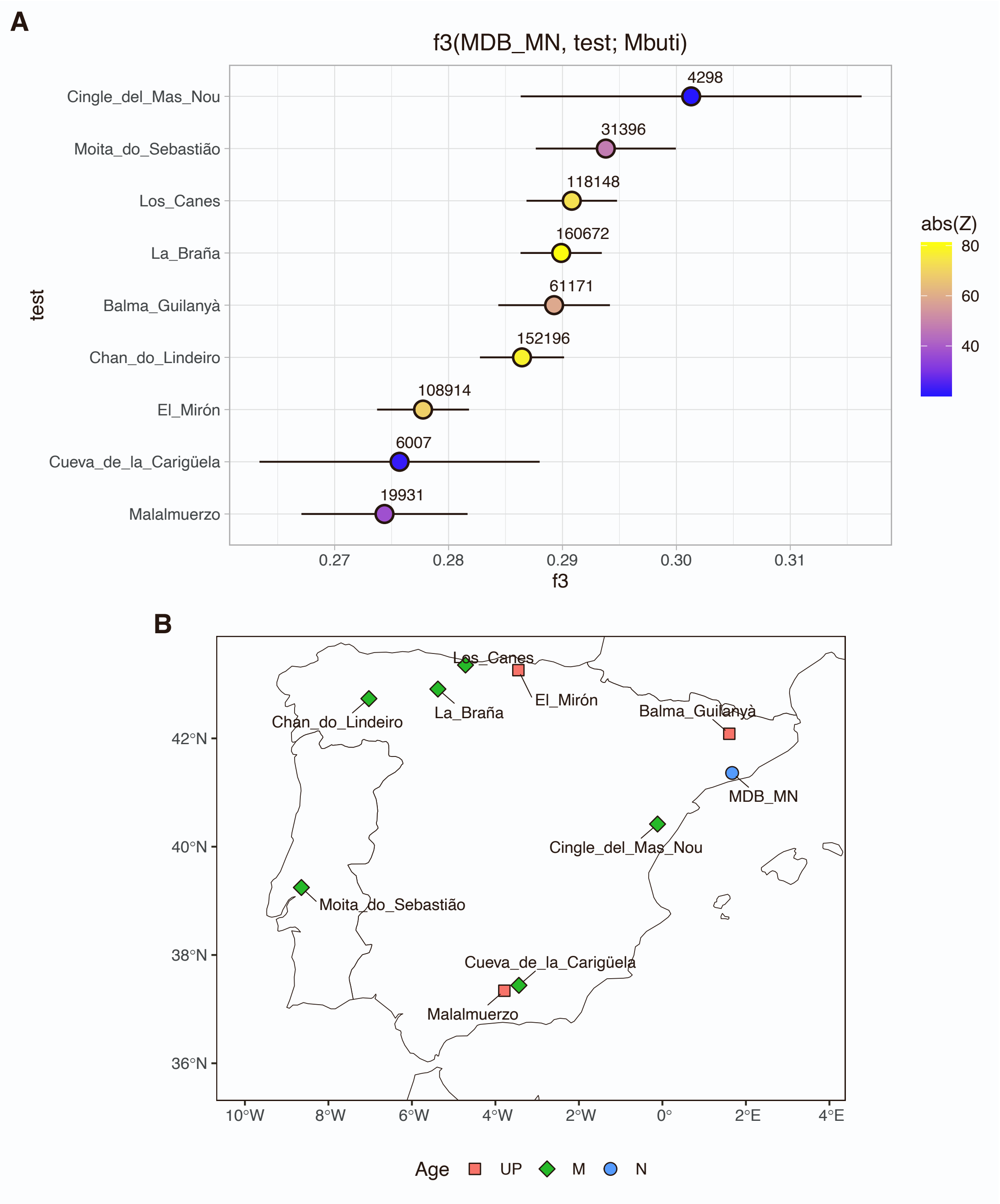

Figure S3. Outgroup f_3-statistics of the form f_3(MDB_MN, Test; Mbuti), where *Test* includes Iberian Upper Palaeolithic and Mesolithic hunter-gatherers. **(A)** The x-axis shows the f_3-statistic values, with results displayed as the mean ± 1-SD and colours representing the Z-scores. The numbers above each dot indicate the number of SNPs used for each calculation. **(B)** Location of the Iberian Upper Palaeolithic and Mesolithic hunter-gatherers used in (A) as well as MDB_MN.

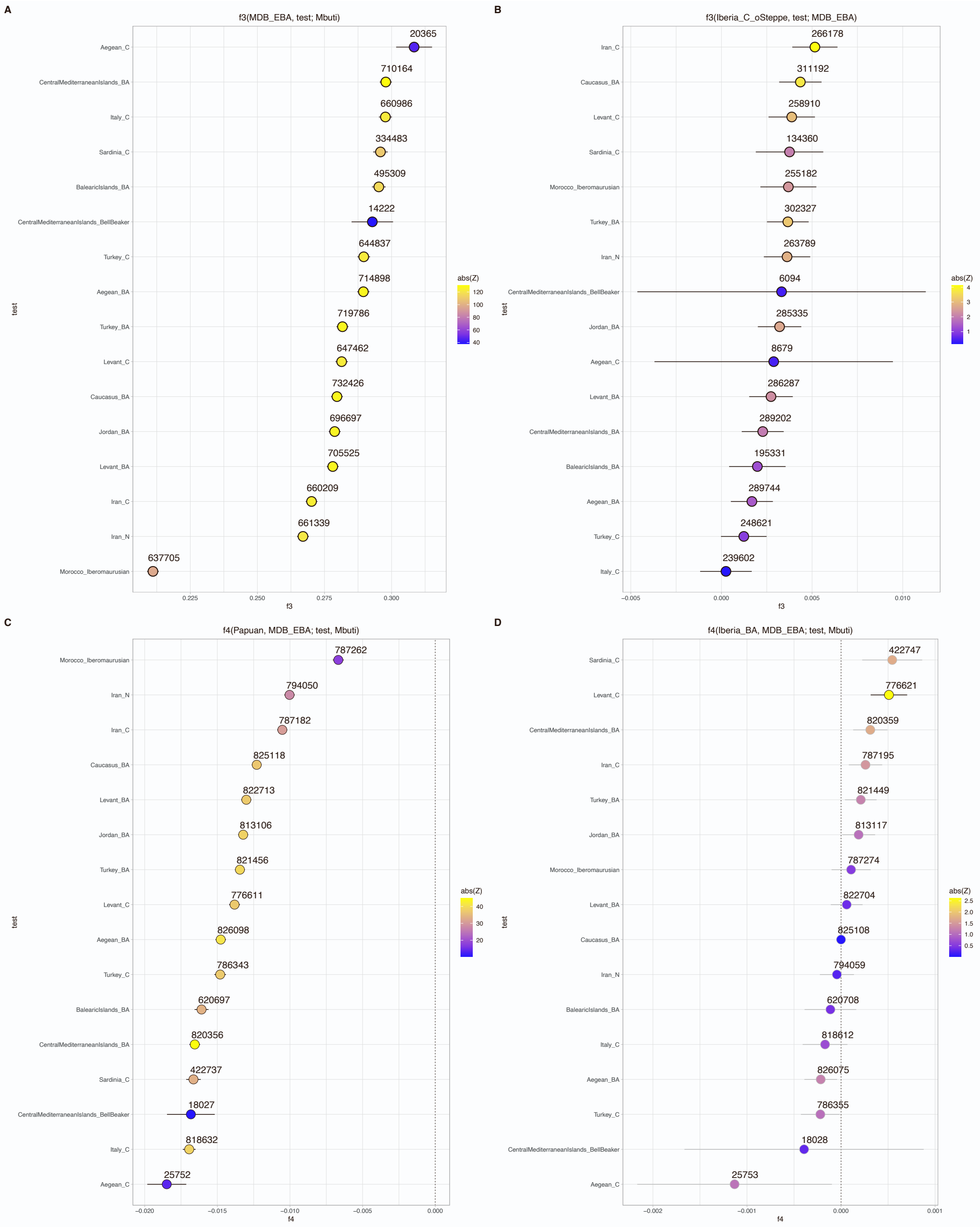

Figure S4. X-axis show different *f*-statistic values and y-axis show different Mediterranean populations. Results are displayed as the mean ± 1-SD and colours represent Z-scores. The numbers above each dot indicate the number of SNPs used for each calculation. **(A)** Outgroup f_3-statistics of the form f_3(MDB_EBA, Test; Mbuti). **(B)** Admixture f_3-statistics of the form f_3(Iberia_C_oSteppe, Test; MDB_EBA). **(C)** f_4-statistics of the form f_4(Papuan, MDB_EBA; Test; Mbuti). **(D)** f_4-statistics of the form f_4(Iberia_BA, MDB_EBA; Test; Mbuti).
